# Patient and Clinician Perspectives on Adolescent Opioid Use Disorder Treatment During a Pandemic: One Step Back, but Two Forward?

**DOI:** 10.2196/23463

**Published:** 2020-10-09

**Authors:** Samuel W Stull, Erin R McKnight, Andrea E Bonny

**Affiliations:** 1 Department of Biobehavioral Health The Pennsylvania State University University Park, PA United States; 2 Division of Adolescent Medicine Nationwide Children's Hospital Columbus, OH United States; 3 Department of Pediatrics The Ohio State University Columbus, OH United States; 4 Center for Clinical and Translational Research The Research Institute at Nationwide Children's Hospital Columbus, OH United States

**Keywords:** adolescent, opioid use disorder, treatment, telehealth, drug, perspective, opioid, COVID-19, young adult

## Abstract

Opioid use disorder (OUD) is one of the most pressing public health problems in the United States and is highly prevalent among adolescents and young adults (AYAs). However, only a small percentage of AYAs with OUD ever receive treatment. Further, among those that do receive treatment, a substantial proportion of patients continue to struggle with OUD, and many prematurely drop out of treatment. These challenges have only been heightened in the face of the COVID-19 pandemic, but greater utilization of telehealth and mobile technologies by OUD patients may help counter these barriers, which ultimately may improve AYA OUD treatment in the postpandemic period. This viewpoint presents the perspective of a person in OUD recovery using online and mobile technology to support his own OUD recovery combined with thoughts from two clinicians supporting AYAs with OUD. Their perspectives may provide insights to help counter COVID-19–related consequences and offer clues to improving AYA OUD treatment in the long term.

## Introduction

Over the last decade, opioid use disorder (OUD) has been among the most pressing public health problems for adolescents and young adults (AYAs) in the United States [1,2]. Now, matters have become even more challenging in the context of the COVID-19 pandemic. Treatment is more difficult to access, widening the already large treatment gap for AYAs with OUD [3]. Fatal overdoses appear to be on the rise likely as a result of social isolation (fewer bystanders available to administer overdose reversal medication) [4]. Furthermore, there is increased risk of severe COVID-19 illness among AYAs with OUD because many are immunocompromised due to smoking, chronic hepatitis, and HIV [5].

Recent changes in the OUD treatment landscape may help counter the negative impact of COVID-19, including federal policy changes decreasing restrictions on buprenorphine prescribing [6] and increased availability of online OUD recovery services. In understanding the implications of these policy-level changes, it is important to garner the perspective of stakeholders on the “ground level,” including clinicians providing OUD treatment and individuals in recovery.

We focus our viewpoint piece on the implications of changes in treatment from in-person to online clinical care, telehealth, and peer support, as well as specific aspects of entering versus maintaining OUD recovery.

## Patient Perspective

My journey on the OUD recovery path, beginning as a young adult, has been built upon frequent contact with in-person social supports. I’ve had the privilege of supportive parents and a sober recovery environment that many AYAs with OUD do not have. I also recognize, however, that I have been consistently one of the youngest members of nearly all the mutual-support recovery meetings I have attended. This reality was only heightened when I re-enrolled in college in a small rural Ohio town, with only a few available meetings and the average age of meeting attendees was nearly 50 years old. Today, during the COVID-19 pandemic, the in-person meetings are certainly missed, but the opportunity to connect with young adults is no longer limited by geography. A plethora of online mutual support recovery meetings exist (worldwide) online. This may not only aid AYAs who are in small numbers at most mutual support recovery meetings, but also any other subpopulation that might be underrepresented.

Of course, there are likely unique benefits to in-person meetings that might otherwise be suboptimal online. For example, accountability to other members in recovery may become less salient or harder to maintain. Recently, an AYA shared in a mutual support meeting that he had gone “cold turkey” and had not “used” for 13 days. He was passing his time walking up and down his block compulsively to distract himself from his cravings and general angst. He stated he would return to join the group again next week…he did not show. Perhaps online meetings lacked the accountability he needed, and in-person meetings would have been better. Alternatively, maybe he would have never attended in-person meetings, and the online format was his first step toward recovery. It is hard to know. For online mutual support, the barriers to recovery support may be lowered, but maintaining recovery may be more challenging in some respects, particularly in terms of accountability.

The timing of OUD recovery, whether initiating or maintaining recovery, may be critical for the usefulness of online versus in-person supports. Early OUD treatment and recovery is complex as one navigates postinpatient treatment and transition to outpatient OUD medications. After inpatient treatment, my transition to outpatient care was complicated by various medication side effects and withdrawal symptoms, requiring frequent communication with my physician. I now realize this was a critical moment in my recovery, where any misstep could have had serious consequences. Initiation and management of OUD medication in the early phases of recovery during COVID-19 may be particularly tricky to navigate. Medication modifications may be needed, providers may not be able to give timely support, and patients may run out of their prescription if they have contracted COVID-19. Now, I also recognize that if online supports were specifically made available for family members supporting those in recovery, it may lower the barriers for them to receive support as well.

Maintaining OUD recovery during COVID-19 has presented me a unique set of challenges. One of the most salient has been the transition from in-person to online recovery support meetings. Social connections with people in OUD recovery have been a mainstay of my own recovery. Online communities have changed the quality of my social interactions in ways that are both helpful and unhelpful. I often feel more comfortable being vulnerable online, partly because the pixelated live stream of my webcam provides a cloak of anonymity. However, social exchanges feel less poignant, lacking a degree of joy, collective empathy, and physical contact. Another ongoing support for recovery I’ve explored is the use of mobile health addiction apps, which offer features ranging from mutual support “meeting locators” to coping and psychological well-being exercises. The potential usefulness of these apps seems particularly important given the lack of in-person resources during the COVID-19 pandemic. However, despite a large increase in the prevalence of these apps over the past few years, I have yet to find one that is consistently useful and engaging. But I am holding out hope that I will find an app that I will use consistently, as some apps are already showing efficacy [7]. Overall, I am hopeful that both online social supports and mobile apps can increase access and support for AYAs in addiction recovery.

## Clinician Perspective

Our experience of collectively providing almost two decades of office-based opioid treatment (OBOT) to AYAs with OUD has been challenged with the onset of the COVID-19 pandemic. Under normal circumstances, initiation of OBOT in an AYA requires a comprehensive in-person evaluation including urine drug screening. However, in light of the extraordinary circumstances presented by the COVID‐19 public health emergency, providers should feel free to prescribe buprenorphine to new patients with OUD following an evaluation via telephone-based voice calls without first performing an in‐person or telemedicine evaluation [6].

Since the onset of Ohio’s shelter-in-place order, our clinic has seen some increase in the number of AYA seeking OBOT services, although our numbers are small. In the 3 months after the shelter-in-place order (April-June), 5 AYAs presented for treatment compared to 2 patients in the 3 months prior (January-March) (historically, we saw no difference in new patient intakes between quarter 1 to quarter 2 during the years 2017-2019). Traditionally, our practice is to perform an in-person initial evaluation with our multidisciplinary team, as well as provide education on medication use, risk of relapse and overdose, and proper use of overdose reversal medications for the patient and support person. A full medical evaluation, including a complete physical examination, serology testing for hepatitis, screening for sexually transmitted infections and HIV are also performed. Although we offered telephone- and video telehealth–based initial evaluations for AYA entering treatment (with the changing insurance landscape, only video telehealth is now available), we have continued to recommend in-person visits, feeling that the benefits outweigh potential risks for most. We have had concerns that the collective social support of the treatment team would be less palpable in a telehealth setting and less likely to alleviate distrust one might have based on previous negative medical interactions. Precautions put in place in the clinical setting to minimize risk include universal masking of patients/visitors, limit of one support person for AYAs <18 years, decreased overall clinic in-person volume, general disinfection/hygiene procedures, and appropriate personal protective equipment for patients and providers based on institutional protocol. To date, encouragement of in-person visits for treatment initiation and the early phases of recovery has not appeared to impact care. Patient engagement with services seems similar to the previous year, with 80% of new patients returning for a follow-up visit post pandemic as compared to 75% in the year prior. However, it is worth considering whether we would have achieved a 100% follow-up rate had we been equally as supportive of telehealth and in-person follow-up for those in the early stages of recovery.

For patients maintaining recovery, we worked to transition them initially to telephone- and now to video telehealth–based care. Although there is concern of potential impact on the lack of urine drug screening accountability, given the safety profile of buprenorphine, the benefits of providing extended prescriptions and refills via telehealth are greater than the risk of severe adverse events like fatal overdoses [8]. To date, all our patients in early remission or sustained recovery (N=35) have remained engaged in treatment, and none have been lost to follow-up. Many patients have found safe, confidential spaces to have their telehealth visits and feel this continues to aid them in their recovery. One patient who struggled to connect during in-person visits, as she needed to bring her children to all appointments, has now been able to engage in lengthy telehealth sessions. However, some still request in-person treatment as the best option for ongoing care, stating that the human connection and increased accountability are beneficial. Furthermore, patients undergoing relapse or those who do not have reliable access to a telephone or video device may also benefit from being seen in person. Patients have been advised to notify us if they are under quarantine to ensure continued access to their medications.

Overall, our initial experience suggests that telehealth is a viable alternative to OBOT for AYA with OUD. Clinic metrics such as return for follow-up visits do not appear to have been negatively impacted. We have been somewhat reluctant to fully embrace telehealth for new patient visits; however, our assumptions on this could be wrong. It is worth exploring whether telehealth treatment initiation increases access without losing any of the benefits of in-person treatment support for those early in recovery.

## Conclusion

Support and management of OUD recovery have been complicated with the arrival of COVID-19. Recent changes in the treatment landscape, including growing telehealth options and decreased restrictions on medication provision, have been directly in response to these current challenges. Although unique considerations may be needed for those entering versus maintaining OUD recovery, from a patient and provider perspective, these changes increase access to care, are helpful elements in the OUD treatment toolbox, and should remain an integral part of treatment after the pandemic.

## Abbreviations

AYA: adolescent and young adult
OBOT: office-based opioid treatment
OUD: opioid use disorder

## References

[1] Gaither JR, Shabanova V, Leventhal JM (2018). US National Trends in Pediatric Deaths From Prescription and Illicit Opioids, 1999-2016. JAMA Netw Open.

[2] Martins SS, Sarvet A, Santaella-Tenorio J, Saha T, Grant BF, Hasin DS (2017). Changes in US Lifetime Heroin Use and Heroin Use Disorder: Prevalence From the 2001-2002 to 2012-2013 National Epidemiologic Survey on Alcohol and Related Conditions. JAMA Psychiatry.

[3] Alinsky RH, Hadland SE, Matson PA, Cerda M, Saloner B (2020). Adolescent-Serving Addiction Treatment Facilities in the United States and the Availability of Medications for Opioid Use Disorder. J Adolesc Health.

[4] (2020). Reports of increases in opioid related overdose and other concerns during COVID pandemic. American Medical Association.

[5] (2019). People Who Are at High Risk for Severe Illness (COVID-19). Centers for Disease Control and Prevention.

[6] (2020). COVID-19-Supporting Access to Buprenorphine. American Society of Addiction Medicine.

[7] Scott CK, Dennis ML, Johnson KA, Grella CE (2020). A randomized clinical trial of smartphone self-managed recovery support services. J Subst Abuse Treat.

[8] Paone D, Tuazon E, Stajic M, Sampson B, Allen B, Mantha S, Kunins H (2015). Buprenorphine infrequently found in fatal overdose in New York City. Drug Alcohol Depend.

